# SSEP N20 and P25 amplitudes predict poor and good neurologic outcomes after cardiac arrest

**DOI:** 10.1186/s13613-022-00999-6

**Published:** 2022-03-15

**Authors:** Sarah Benghanem, Lee S. Nguyen, Martine Gavaret, Jean-Paul Mira, Frédéric Pène, Julien Charpentier, Angela Marchi, Alain Cariou

**Affiliations:** 1 Medical ICU, Cochin Hospital, AP-HP, 27 rue du Faubourg Saint-Jacques, 75014 Paris, France; 2 Medical School, University of Paris, Paris, France; 3 CMC Ambroise Paré, Research and Innovation, Neuilly-sur-Seine, France; 4 Neurophysiology Department, GHU Psychiatrie et Neurosciences, Sainte Anne Hospital, Paris, France; 5 After ROSC Network, Paris, France; 6 Paris-Cardiovascular-Research-Center (Sudden-Death-Expertise-Center), INSERM U970, Paris, France; 7 INSERM 1266, Institut de Psychiatrie et Neurosciences de Paris-IPNP, Sainte Anne Hospital, Paris, France

**Keywords:** Cardiac arrest, Prognosis, Persistent coma, Neuroprognostication, Somato sensory evoked potential, EEG, NSE, Status myoclonus

## Abstract

**Background:**

To assess in comatose patients after cardiac arrest (CA) if amplitudes of two somatosensory evoked potentials (SSEP) responses, namely, N20-baseline (N20-b) and N20–P25, are predictive of neurological outcome.

**Methods:**

Monocentric prospective study in a tertiary cardiac center between Nov 2019 and July-2021. All patients comatose at 72 h after CA with at least one SSEP recorded were included. The N20-b and N20–P25 amplitudes were automatically measured in microvolts (µV), along with other recommended prognostic markers (status myoclonus, neuron-specific enolase levels at 2 and 3 days, and EEG pattern). We assessed the predictive value of SSEP for neurologic outcome using the best Cerebral Performance Categories (CPC1 or 2 as good outcome) at 3 months (main endpoint) and 6 months (secondary endpoint). Specificity and sensitivity of different thresholds of SSEP amplitudes, alone or in combination with other prognostic markers, were calculated.

**Results:**

Among 82 patients, a poor outcome (CPC 3–5) was observed in 78% of patients at 3 months. The median time to SSEP recording was 3(2–4) days after CA, with a pattern “bilaterally absent” in 19 patients, “unilaterally present” in 4, and “bilaterally present” in 59 patients. The median N20-b amplitudes were different between patients with poor and good outcomes, i.e., 0.93 [0–2.05]µV vs. 1.56 [1.24–2.75]µV, respectively (*p* < 0.0001), as the median N20–P25 amplitudes (0.57 [0–1.43]µV in poor outcome vs. 2.64 [1.39–3.80]µV in good outcome patients *p* < 0.0001). An N20-b > 2 µV predicted good outcome with a specificity of 73% and a moderate sensitivity of 39%, although an N20–P25 > 3.2 µV was 93% specific and only 30% sensitive. A low voltage N20-b < 0.88 µV and N20–P25 < 1 µV predicted poor outcome with a high specificity (sp = 94% and 93%, respectively) and a moderate sensitivity (se = 50% and 66%). Association of “bilaterally absent or low voltage SSEP” patterns increased the sensitivity significantly as compared to “bilaterally absent” SSEP alone (se = 58 vs. 30%, *p* = 0.002) for prediction of poor outcome.

**Conclusion:**

In comatose patient after CA, both N20-b and N20–P25 amplitudes could predict both good and poor outcomes with high specificity but low to moderate sensitivity. Our results suggest that caution is needed regarding SSEP amplitudes in clinical routine, and that these indicators should be used in a multimodal approach for prognostication after cardiac arrest.

**Supplementary Information:**

The online version contains supplementary material available at 10.1186/s13613-022-00999-6.

## Introduction

A vast majority of patients resuscitated from cardiac arrest (CA) are comatose after return of spontaneous circulation (ROSC) because of anoxic–ischemic brain injury. A significant number of patients remain unconscious after rewarming from targeted temperature management (TTM) and discontinuation of sedation. Despite the improvement of post CA care, most of them will die following withdrawal of life-sustaining treatment (WLST) for irreversible post-anoxic encephalopathy [1]. Early identification of prognosis is one of the most challenging issues in this situation. The European Resuscitation Council and European Society of Intensive Care Medicine guidelines recommend initiating WLST based on prognostication of a poor neurological outcome [2, 3]. It is, therefore, essential to minimize the risk of a falsely pessimistic prediction. The recommended strategy is to apply a multimodal prognostication approach combining clinical examination, neurophysiological investigations (electroencephalogram (EEG); somatosensory evoked potentials (SSEPs)), biological (Neuron specific enolase, NSE), and neuroradiological (CT scan or MRI) tools. This prognostication strategy algorithm is recommended in comatose patients defined as motor component of the GCS lower or equal to 3 at 72 h after ROSC, in the absence of confounders, in particular residual sedation. Sedative drugs weakly influence SSEP, unlike their influence on the EEG [4, 5]. Bilateral absence of N20 SSEP responses is one of the most specific elements for poor outcome forecast, reflecting the primary somato-sensory cortex and the thalamo-cortical loop injuries [6, 7]. Nevertheless, the sensitivity of N20 bilateral absence is relatively low, reaching approximatively 30% [2, 8]. To enhance SSEP sensitivity, some recent studies explore the role of cortical SSEP N20 amplitude, defined as the peak-to-peak amplitude between the N20 (i.e., the negative deflection 20 ms after stimulation) and P25 (i.e., the positive deflection 25 ms after stimulation) responses [9–12]. Using different cutoff values, a low N20 voltage was reported to improve sensitivity for prediction of poor outcome as compared with the dichotomous classification (presence or absence of N20 latency) from 46 to 47% [11], 30% to 57% [10], and 53 to 73% [12]. On the other hand, the interest of N20 amplitude for good outcome prediction remains poorly explored and provided conflicting results. No correlation was found between N20 amplitude and outcome in a recent study [11] although another study reported that N20 amplitude > 3 μV could predict good outcome with a sensitivity of 61% and a FPR of 11% [12]. Moreover, the interest of N20-baseline amplitude has been poorly explored [10]. Finally, these studies accorded limited attention to the correlation to other prognostic markers [12].

The aim of the present study was to assess the prognostic value of two SSEP responses, i.e., N20-baseline and N20–P25 amplitudes for prediction of neurologic outcome after CA, and to assess the correlation between SSEP amplitudes and other prognostics markers.

## Methods

### Population

All consecutive adult patients who were admitted between November 2019 and July 2021 to the intensive care unit (ICU) of Cochin University Hospital (Paris, France) in a comatose state (defined as a Glasgow coma scale [GCS] ≤ 8 with a GCS motor < 3 and a Richmond Agitation–Sedation Scale RASS ≤ -4) after resuscitation from CA, regardless of initial rhythm, with SSEP performed, were prospectively considered for inclusion. We excluded patients investigated for brain death diagnosis, patients awake before SSEP, and patients who died within 48 h post CA, before a reliable neurological examination could be performed. Patients’ next of kin were informed that data were collected for clinical research purposes.

### Data collection

The following data were recorded: patients' characteristics, pre-hospital care and cardiac arrest management data using Utstein style, in-hospital variables including serum lactate at admission, TTM use, type of sedation, clinical indicators of neurological status (clinical status myoclonus defined according to ERC/ESICM guidelines as generalized, continuous and persisting for 30 min or more of the myoclonic jerks, requiring an anti-epileptic drugs regardless EEG results), EEG patterns, SSEP recording and NSE levels [13]. In the present analysis, we used the first EEG and SSEP performed during the ICU stay; NSE levels were determined at days 1, 2 and 3 after CA. Length of stay and cause of death were also reported. Data collection was approved by the Ethics Committee of the French Intensive Care Society (#CESRLF_12-384 and 20–41) and conducted according to French health authorities’ regulations (French Data Protection Authority #MR004_2209691).

### ICU management

The management protocol for patients admitted to our ICU after CA reported in Additional file 1: ESM1 has been previously described and did not change throughout the study period [2, 3, 14]. In the absence of contraindication, TTM was immediately started after ICU admission with a target temperature of 32–36 °C adapted to hemodynamic tolerance and using an external cooling device for 24 h. Sedation protocol, according to guidelines, used short acting drugs including propofol and remifentanil. Sedation protocol was based on the RASS, titrated to obtain a RASS of − 5 (no response to voice or physical stimulation) and was interrupted after rewarming (Additional file 1: ESM1).

### Neurological prognostication and WLST

Neurological status was evaluated every 3 h by nurses, and daily by ICU physicians until death or ICU discharge. Awakening was defined as three consecutive RASS scores of at least − 2 (patient briefly awakens with eye contact to voice), as previously reported [15]. In patients who were still comatose 72 h after ROSC and 48 h after sedation discontinuation, a multimodal prognostication protocol was used, unchanged during study period, consistent with the 2015 international ERC/ESICM guidelines [13]. WLST was considered in comatose patients with a GCS motor score 1 or 2 when two or more of the following conditions were present: (1) bilaterally absent pupillary and corneal reflex; (2) bilaterally absent N20 waves on SSEP; or (3) refractory electrical status epilepticus, burst suppression or suppression. Electrical status epilepticus was defined as refractory when it did not improve after treatment with 2 lines of major antiepileptic drugs (among phenytoin, phosphenytoin, valproate, phenobarbital). Amplitudes of SSEP was not used in our prognostication algorithm and did not influence WLST (Additional file 1: ESM2).

### SSEP recording

SSEP recordings were made using Deltamed Coherence (Natus, Middleton, USA). SSEP were recorded in patients still comatose 72 h after ROSC and 48 h after sedation discontinuation. The SSEP was measured after stimulation of the right and left median nerve using a bipolar surface electrode at the wrist. Stimulation intensity was adjusted to produce visible thumb twitches; if neuromuscular blocking agents were administered, Erb amplitudes were used instead. Monophasic rectangular-wave 200 ms stimulus pulses were delivered. Stimulus frequency was set at 2–3 Hz. Poststimulus recording lasted 50 ms (bandwidth: 30 Hz–3 kHz, sampling frequency: 50 kHz). Two or three sets of 300–1000 responses were averaged. Surface electrodes were positioned at Erb’s points. Needle electrodes were used for scalp derivations: 2 cm posterior to C3 and C4 (C3’ and C4’). N9 (peripheral), N13 and N20 (cortical) responses were recorded. N9 peripheral responses corresponded to Erb's point ipsilateral to the stimulation vs. reference electrode at contralateral Erb's point. For cortical responses, a bi-parietal montage (active electrode contralateral to the stimulation vs. reference electrode ipsilateral to the stimulation) was used.

### SSEP interpretation

**Fig. 1:**
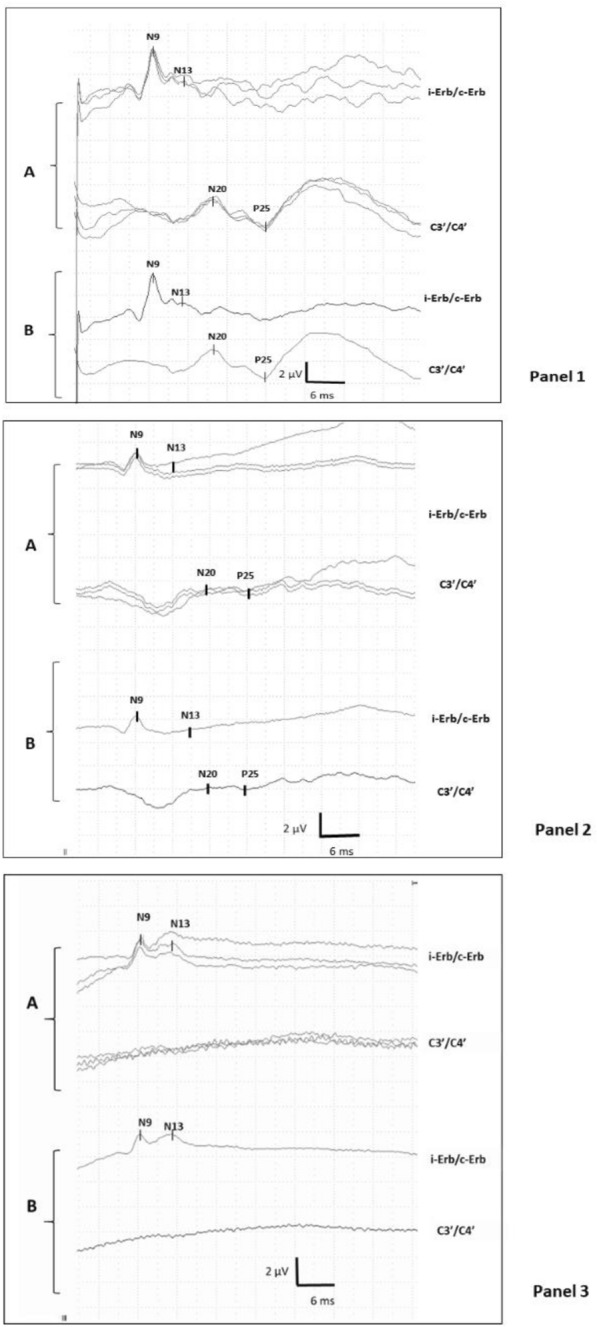
Two channels median SSEPs after stimulus of the right median nerve. In a normal SSEP (panel 1), the channels show the cortical responses N20 and P25, the spinal component (N13) and the peripheral component (N9). In the panel 2, N20 and P25 were presented but amplitudes were reduced. In the panel 3, N20 and P25 were absent; **A** superposed Evoked Potentials; **B** averaged Evoked Potentials. *i-Erb* ipsilateral Erb’s point, *c-Erb* contralateral Erb’s point

N20 latencies were deemed interpretable if at least 2 peripheral (N9), 2 spinal peak (N13) and cortical recordings (N20–P25) per side were bilaterally reproducible and if a noise level below 0.25 μV in all 4 cortical recordings had been achieved. Noise level was determined 5–10 ms after stimulation to exclude stimulation artifacts. The noise level was determined automatically and visually checked. Digitalized SSEPs were reevaluated blinded to patients’ outcome by an expert electrophysiologist. Figure 1 shows representative examples of SSEP recordings. The N20 wave was identified as the major negative peak (C’3–C’4 montage), while P25 was identified as the major positive peak following N20. Three profiles of SSEP responses were determined according to the presence or absence of N20 response. In patients with no reproducible cortical potential and noise level below 0.25 μV, the recording was classified as «absent-absent/AA». Patients with reproducible cortical potential on both sides were classified as «present present/PP». If there was only a unilateral response, we classified patients as «absent-present/AP». As “AA” pattern was already recognized as a robust marker for poor neurological outcome, we only assessed SSEP amplitudes in case of bilateral responses defined as «PP» patterns. We defined N20-baseline amplitude as the highest difference between N20 peak and the baseline and the N20–P25 amplitude using the peak-to-peak N20–P25 amplitude. Amplitude computation was automated by the analysis software use. In case of asymmetry between right and left N20, we retained the best amplitude value. For prediction of poor outcome, we considered the patterns «AA» and «PP», while for prediction of good outcome, we only considered the «PP» pattern. Concerning the Deltamed device normal SSEP amplitudes, the median N20–baseline and N20–P25 amplitudes were 1.89(1.2–2.55)µV and 2.05(1.29–2.55)µV, respectively.

### Electroencephalogram (EEG)

EEG recordings were acquired maximum 1 h before SSEPs’ recording over 20 to 30 min with a Natus Deltamed recording system (Natus, Middleton, USA) using 19 electrodes placed according to the 10–20 international system, with additional ground and reference electrodes. During the recordings, auditory (calling of patient’s first name and family name, hand clapping), somatosensory and pain stimuli were applied at least twice, with a minimum of 10 s intervals. EEG traces were retrospectively analyzed de novo by one board-certified electroencephalographers (AM) blinded to the clinical outcome and the SSEPs results. EEGs were interpreted according to the standardized criteria of the American Clinical Neurophysiology Society [16]. Each EEG was classified into one of the mutually exclusive categories defined by Westhall et al. [17], namely, highly malignant pattern (suppressed background or burst-suppression, with or without superimposed periodic pattern), malignant pattern (presence of at least one of the following: abundant periodic discharges or rhythmic spike-waves, electroencephalographic seizure, discontinuous or low-amplitude background, reversed anterior–posterior amplitude gradient, absence of reactivity) or benign pattern (continuous and reactive pattern, absence of malignant feature) [16, 17].

### Outcome assessment

The primary outcome was the neurological status at 3 months using the “best” cerebral performance categories (CPC) scale (Additional file 1: ESM3). CPC is defined as CPC1 = good cerebral performance no or minimal disability; 2 = moderate cerebral disability, i.e., disabled but independent; 3 = severe cerebral disability, i.e., conscious but disabled and dependent; 4 = comatose or vegetative state; 5 = death [2, 18]. Good neurological outcome was defined as CPC 1–2, using structured phone interviews. We used the best CPC to avoid considering patients classified as CPC1–2 and who subsequently died (CPC5) from non-neurological causes as poor neurological outcome (CPC3–4–5).

The secondary outcome was the best CPC at 6 months. Finally, correlation of SSEP amplitude with NSE peak, EEG patterns and clinical status myoclonus was assessed.

### Statistical analysis

Continuous variables were summarized using medians and interquartile range (IQR), and categorical variables were reported as proportions. We performed Pearson’s Chi2 test for categorical variables, and Wilcoxon, when appropriate, for continuous variables. We assessed the association between candidate variables including patient demographics, Utstein variables, status myoclonus, SSEP recording, NSE peak at D2 and D3 and EEG patterns; regarding neurologic outcome for which we used a binary outcome (CPC 1–2 vs. 3–4–5).

N20-baseline and N20–P25 SSEP amplitudes thresholds were sought in patients with “PP” cortical responses, setting for the best compromise between the higher specificity (and so the lower FPR), associated with an acceptable sensitivity [19]. All statistical analyses were performed using IBM SPSS version 26.0 (IBM Corporation, Armonk, New York). A two-sided *p* value < 0.05 was considered to be statistically significant. Figures were drawn using Prism 8.4.3 (GraphPad Software, California, USA) and R software 3.6 (R project, worldwide community software).

## Results

### Patients

**Fig. 2:**
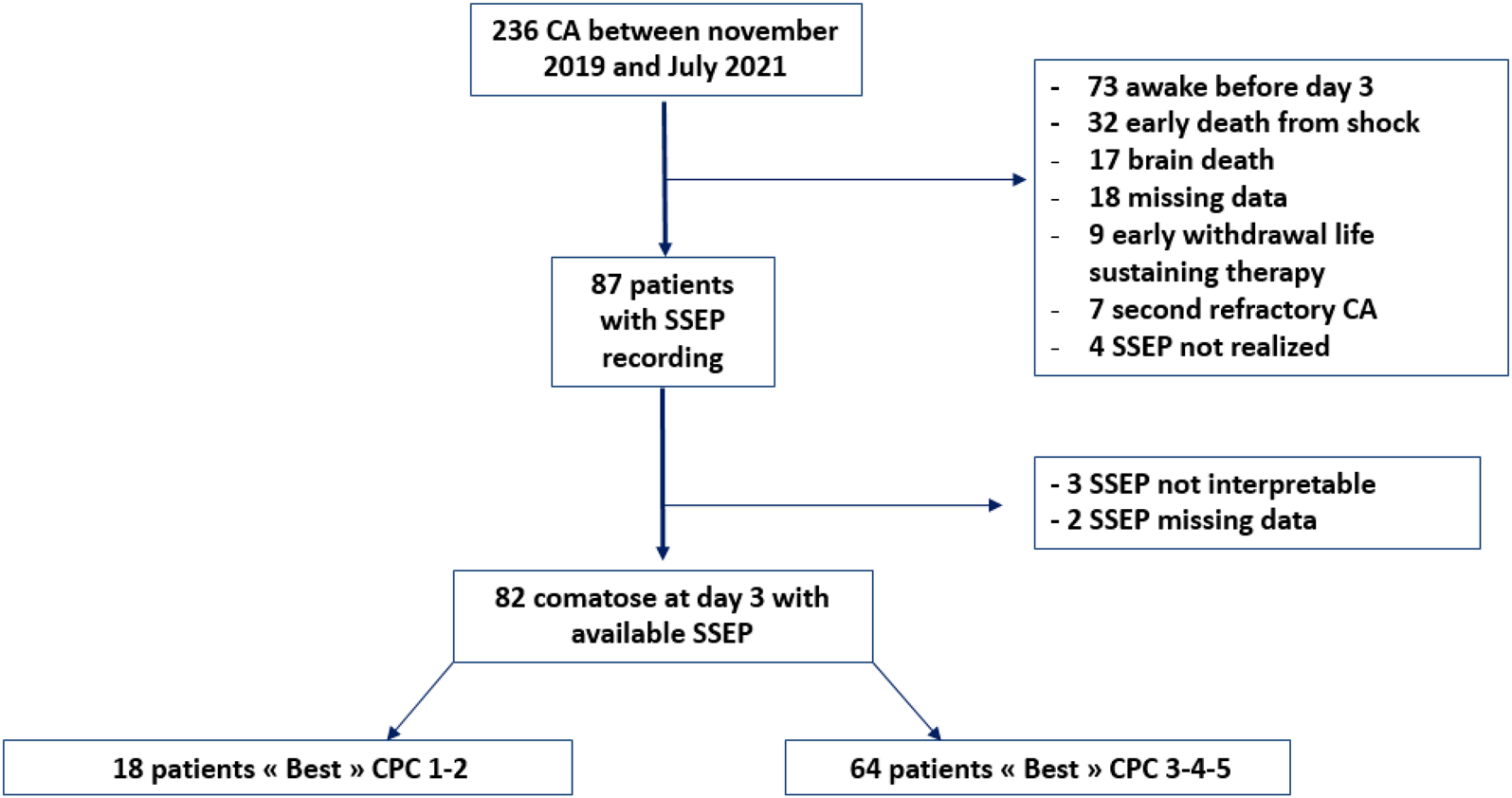
Flow chart

From November 2019 to July 2021, 236 patients were admitted after CA, among which 87 were comatose at day 3 after CA, and had at least one SSEP performed during their ICU stay. Among them, 5 patients were excluded: 3 recordings were considered as not interpretable (absent N9 and/or important noise) and 2 patients presented SSEP missing data (Flow chart Fig. 2).

Baseline characteristics are described in Table 1 for the 82 patients who were retained in the analysis. Patients were mostly male (61%), with median age of 62(50–75) years. Initial rhythm was shockable in 30.5%. TTM was used in 79% of patients, with propofol infusion in all cases. A poor outcome was observed in 64/82 patients (78%) at 3 and 6 months. Causes of death were mainly due to WLST in 55/64 (86%) patients, presumably secondary to an irreversible post anoxic encephalopathy.

**Table 1:** Patient’s characteristics

	CPC 3–4–5 *n* = 64	CPC 1–2 *n* = 18	*p*
Male gender, n (%)	41 (64%)	10 (55,6%)	0.59
Age, years, median (IQR)	66.5 (51–76)	62 (54–71)	0.73
CA in a public area, n (%)	21 (33%)	8 (44%)	0.52
Bistander CPR, n (%)	49 (76%)	17 (94%)	0.17
Initial shockable rythm, n (%)	16 (25.4%)	12 (66.7%)	0.0019
No flow, min, median (IQR)	2 (0–5)	0 (0–3)	0.058
Low flow, min, median (IQR)	21,5 (15–26.5)	16 (8–21)	0.01
TTM, n (%)	48 (76%)	17 (94.4%)	0.10
Sedation with Propofol/remifentanil, n (%)	64 (100%)	18 (100%)	1
ICU length of stay, days, median (IQR)	7 (5–11.5)	13 (7–23)	0.02

### SSEPs findings

SSEP recordings were obtained at a median time of 3[2–4] days after CA (Table 2). Thus, SSEP amplitudes could be determined in all patients (Fig. 2). Regarding SSEP patterns, 59 patients presented a «PP» pattern, 4 patients a «AP» pattern and 18 patients a «AA» pattern. The scatter plot shows the distribution of the N20-baseline and N20–P25 amplitudes according to the five CPC categories at 3 months and 6 months (Additional file 1: ESM4). The median N20-baseline amplitude was 1.56 [1.24–2.75]µV in patients with favorable outcome and 0.93 [0–2.05]µV in poor outcome group (*p* = 0.008). The median N20–P25 was 2.64 [1.39–3.8]µV and 0.57 [0–1.43]µV in good and poor outcome patients, respectively (*p* < 0.0001). The median SSEP amplitudes of patients with a “AP” pattern are reported in Additional file 1: ESM5. Others prognostic markers are reported in Table 2.

**Table 2:** Prognostic elements stratified for outcome within 3 months

* n* = 82	CPC3–4–5 *n* = 64	CPC1–2 *n* = 18	*p*
CA-SSEP delay, days, median (IQR)	3 (2.5–4)	3 (2–4)	0.62
SSEP responses:			0.01
Bilaterally absent «AA»	19 (30%)	0	
Unilaterally present «AP»	4 (6%)	0	
Bilaterally present «PP»	41 (64%)	18 (100%)	
N20-Baseline amplitude (µV) of the «PP» patterns, median	0.93 (0–2.05)	1.56 (1.24–2.75)	0.008
N20–P25 amplitude (µV) of the «PP» patterns, median MD n = 3	0.57 (0–1.43)	2.64 (1.39–3.80)	< 0.0001
NSE peak at day 2, median (µg/l)	96.5 (46–240)	29.5 (20–42)	< 0.0001
NSE peak at day 3, median (µg/l)	157 (54–353)	21 (16–36)	< 0.0001
Highly malignant EEG, n (%)	23 (34.4%)	0 (0%)	0.0021
Malignant EEG, n (%)	32 (50%)	2 (11%)	0.0029
Benign EEG, n (%)	9 (14%)	16 (89%)	< 0.0001
Status myoclonus n (%)	36 (56.3%)	1 (5.6%)	< 0.0001

*CA* cardiac arrest, *EEG* electroencephalogram, *MD* missing data, *NSE* neurone specific enolase

### Correlation between SSEPs amplitudes and prognostic biomarkers

**Fig. 3:**
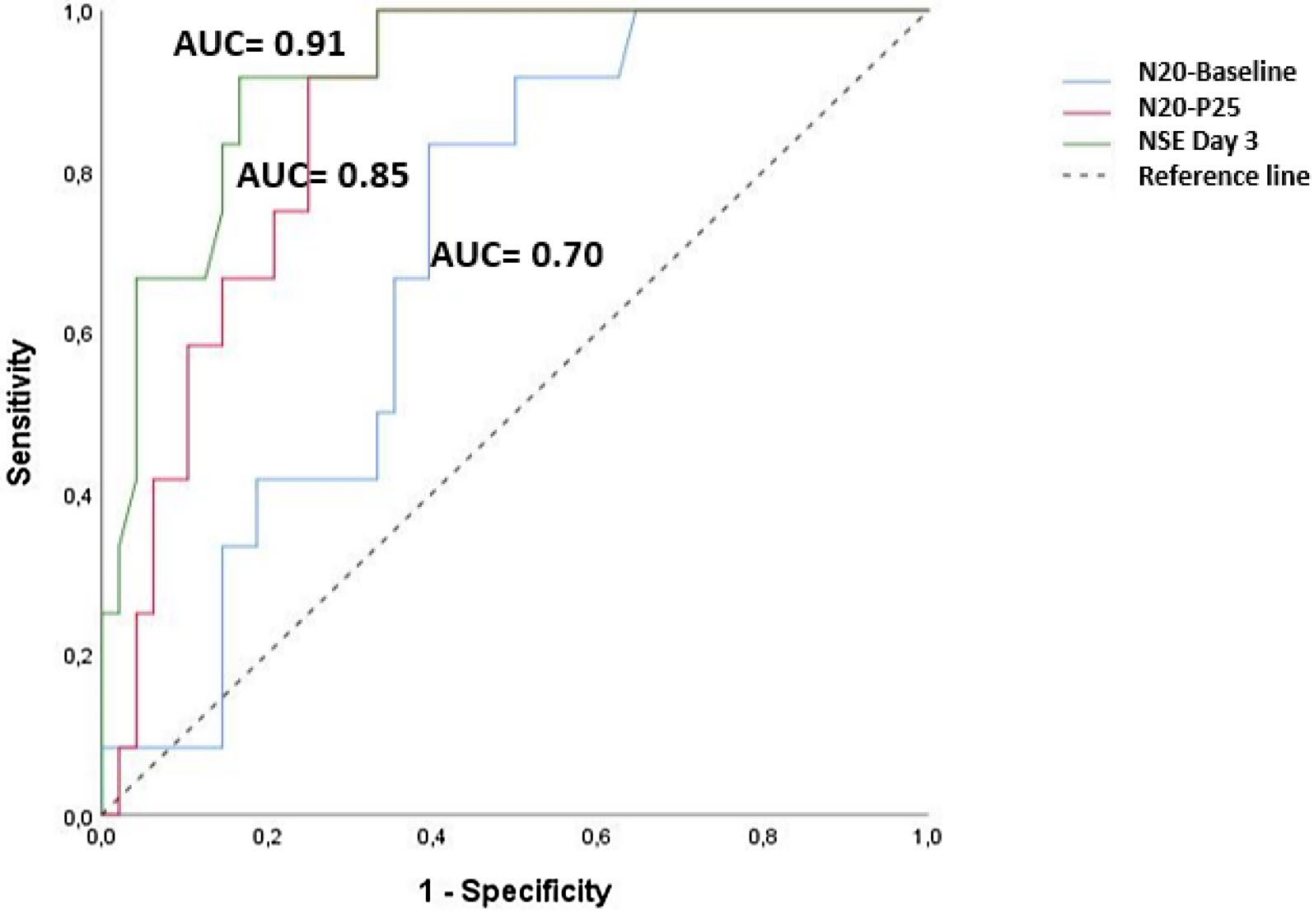
The receiver operating characteristic (ROC) curves for CPC at 3 months showing the predictive powers of SSEP amplitudes and NSE peak at day 3 after CA. Respective ROC area under curves (AUC) are: NSE at day 3= 0.91 (0.85–0.98), *p* < 0.0001; N20-P25=0.85 (0.76–0.94), *p* < 0.0001; N20-baseline=0.70 (0.58-0.81), *p* = 0.012

N20-baseline and N20–P25 amplitudes was significantly correlated to demographic characteristics and prognostic biomarkers as we found a negative correlation with low flow and NSE peak > 60 µg/ml at day 2 and day 3 after CA and a positive correlation between N20–P25 amplitude and a benign EEG (Additional file 1: ESM6). Despite this, ROC analysis revealed that the predictive performance of the serum NSE peak at day 3 was good with an AUC value of 0.91 [0.85–0.98], *p* < 0.0001, although the AUC value of N20-baseline and N20–P25 amplitudes were 0.70 [0.58–0.81], *p* = 0.012 and 0.85 [0.76–0.94], *p* < 0.0001, respectively (Fig. 3).

### Prediction of good outcome

All patients with an N20-baseline > 4.6 µV presented a good outcome, with a specificity of 100 [100–100]% but a sensitivity of 5 [3–7]%. Lowering the N20-baseline threshold to > 2.7 µV or 2 µV increased the sensitivity to 28 [23–33]% and 39 [33–44]% with a specificity of 87 [83–91]% and 73 [68–78]%, respectively (Table 3). Regarding the second SSEP component i.e N20–P25, an amplitude > 3.2 µV predicted good outcome with a specificity of 93 [90–96]% and sensitivity of 29 [23–34]%, although a threshold of 4 µV increased specificity (sp = 95 [92–97] %) but decreased sensitivity (se = 14 [10–18]%) (Table 3).

**Table 3:** Accuracy of the different thresholds SSEP, EEG benign patterns, no myoclonus, NSE peak at day 3 < 60 µg/ml, and combinations for prediction of good outcome

	Se(%)	Sp(%)	FPR(%)
N20-baseline > 2 µV	39 (33–44)	73 (68–78)	27 (22–32)
N20-baseline > 2.7 µV	28 (23–33)	87 (83–91)	13 (9–17)
N20–P25 > 3,2 µV	29 (23–34)	93 (90–96)	7 (4–10)
N20–P25 > 4 µV	14 (10–18)	95 (92–97)	5.4 (3–8)
No status myoclonus	94 (92–97)	56 (51–62)	44 (38–49)
Day 3 NSE peak < 60 µg/ml	93 (90–96)	74.5 (69–80)	25.5 (20–31)
Benign EEG	89 (85–92)	86 (82–90)	14 (10–18)
N20-baseline > 2 µV + benign EEG	33.3 (28–39)	96.9 (95–99)	3.1 (1–5)
N20-baseline > 2 µV + NSE < 60 ng/ml	28 (23–33)	90.6 (87–94)	9.4 (6–13)
N20-baseline > 2 µV + no status myoclonus	33.3 (28–39)	86 (82–90)	14 (10–18)
N20–P25 > 3.2 µV + benign EEG	17 (13–21)	97 (93–98)	3 (2–7)
N20–P25 > 3.2 µV + day 3 NSE < 60 µg/ml	17 (13–21)	98.4 (97–100)	1.6 (0–3)
N20–P25 > 3.2 µV + No status myoclonus	17 (13–21)	95.3 (93–98)	4.7 (2–7)

The combination of N20-baseline > 2 µV with a benign EEG presented a higher specificity (sp = 96.9 [95–99]%) with a similar sensitivity (se = 33.3 [28–39]%) although combination of N20–P25 with benign EEG was also highly specific (sp = 97 [93–98]%) but poorly sensitive (se = 17 [13–21]%). Prognostic value of benign EEG, NSE and others combinations of markers are reported in Table 3 and Additional file 1: ESM7.

### Prediction of poor outcome

An N20 baseline < 0.35 µV predicted poor outcome with 100 [100–100]% specificity and 35 [31–39]% sensitivity. Increasing this threshold to < 0.88 µV improved the sensitivity to 50 [44–56]%, while specificity was 94 [92–97]%. An N20–P25 < 1 µV seemed to be the best compromise between a high specificity (sp = 93 [90–96]%) and an acceptable sensitivity (se = 66 [60–72]%)(Table 4). SSEPs were bilaterally absent in 19 patients. Considering subjects with absent or low voltage SSEP, sensitivity increased from 30% for the «bilaterally absent» pattern alone to 58 [52–63]% for the «bilaterally absent or N20–P25 < 1 µV» pattern (*p* = 0.002) and to 47 [41–52]% for the «bilaterally absent or N20-baseline < 0.88 µV» pattern (*p* = 0.01). Prognostic value of malignant EEG, NSE and other combinations of markers are reported in Table 4 and Additional file 1: ESM7.

**Table 4:** Accuracy of the SSEP and EEG malignant patterns, status myoclonus, NSE > 60 µg/ml and combinations for prediction of poor outcome

	Se(%)	Sp(%)	FPR(%)
N20-baseline < 0,35 µV	35 (31–39)	100 (100–100)	0 (0–0)
N20-baseline < 0,88 µV	50 (44–56)	94 (92–97)	6 (3–8)
N20–P25 < 0,56 µV	50 (44–56)	100 (100–100)	0 (0–0)
N20–P25 < 1 µV	66 (60–72)	93 (90–96)	7 (4–10)
Status myoclonus	56 (51–62)	94 (92–97)	6 (3–8)
Day 3 NSE peak > 60 µg/ml	25 (20–31)	93 (90–96)	7 (4–10)
Malignant EEG	50 (44–56)	89 (85–92)	11 (8–15)
N20-baseline < 0,88 µV + NSE > 60 µg/ml	36 (31–41)	100 (100–100)	0 (0–0)
N20–P25 < 1 µV + NSE > 60 µg/ml	44 (38–49)	100 (100–100)	0 (0–0)
Bilaterally absent N20 SSEP	30* (25–35)	100 (100–100)	0 (0–0)
N20-baseline < 0.88 µV or N20 bilaterally absent	47 (41–52)	94 (92–97)	6 (3–8)
N20–P25 < 1 µV or N20 bilaterally absent	58* (52–63)	94 (92–97)	6 (3–8)

^*^p = 0.002

## Discussion

In patients still comatose 72 h after CA, we found that the N20-baseline and N20–P25 SSEPs’ amplitudes could be used to predict both good and poor outcome. For good outcome prediction, we found that a threshold of N20-baseline > 2 µV and N20–P25 > 3.2 µV recording at 72 h after CA was the best compromise between a high specificity and acceptable sensitivity. Regarding poor outcome prediction, a threshold of 0.88 µV for N20-baseline and 1 µV for N20–P25 amplitudes seemed to be the best compromise. Second, we also highlighted that SSEP amplitudes correlate with other prognostic variables, namely, NSE peak evaluated at days 2 and 3. Finally, we highlighted that the sensitivity of the “bilaterally absence or low voltage SSEP” was significantly higher than that of the “bilateral absence SSEP” alone (58 vs. 30%, *p* = 0.002).

Our results are in agreement with previous data. The prognostic value of high SSEP amplitude for good outcome prediction has been described with different thresholds. In these studies, cutoff values > 2.5 µV [10], > 3 µV [12] and > 3.6 [20, 21] were considered as the best compromise between a low FPR and a high sensitivity. In a recent postmortem study that assessed prognostic markers in conjunction with the histopathological severity of hypoxic–ischemic encephalopathy (HIE) obtained from autopsy, the severity of HIE increased with decreasing N20 amplitude, and an N20 amplitude > 2.5 µV was associated with an absence of severe HIE [22]. Regarding poor outcome prediction, our results are also in agreement with those previously described, namely, < 1.01 µV [12], < 0.64 µV [9, 23] and < 0.41 µV [11]. These threshold differences between studies could be due first to the different timing of N20 assessment (i.e., at 12–48 h after CA in previous studies), to the different definitions of the N20 amplitudes, requiring a standardization of the evaluation to use the N20 amplitude as a prognostic marker and finally to the subjective definition by physicians of the “best compromise” between a high specificity and an acceptable sensitivity.

Although there is no recommendation yet regarding the systematic reporting of the cortical amplitude rather than the binary response “absent/present” SSEP, and no recommendation about the N20–P25 or N20-baseline components use [2, 4], our results suggest that caution is needed regarding N20 SSEP amplitudes in routine practice. The underlying concept is that the amplitude of the N20-baseline and N20–P25 is inversely related to the severity of neurological injury, and that these amplitudes could be assessed as a continuum rather than a categorical variable [24]. Moreover, the interrater reliability of dichotomized SSEP evaluation as absent of present may be variable among expert neurophysiologists, especially at noise levels > 0.25 µV, and an assessment as a continuous variable could improve this point [25].

Unlike EEG, SSEPs are considered as largely unaffected by the timing of SSEP recording mainly assessed at 48–72 h after CA, the body temperature, or sedation infusion. On the other hand, EEG pattern and serum NSE might display a greater discriminating value in patient’s prognostication as compared with SSEPs. In fact, SSEP can be identified even in the presence of highly malignant EEG, considered as a robust indicator of poor outcome [26, 27]. A potential explanation for this is that the SSEP generator is localized on the somatosensory cortex, receiving inputs from the thalamus. Thus, SSEP responses do not reflect the complex cortical neuronal interplay of the EEG signal. This also could explain why unresponsive wakefulness/vegetative state patients present preserved SSEP responses, due to the dysfunction of the default mode network, but not of the primary sensory cortex related to SSEP generation [7, 8]. In this context, we chose to evaluate the prognostic value of N20-baseline and N20–P25 responses, because cortical generators of N20 and P25 could be different [9]. Indeed, N20 and P25 peaks are widely accepted to be generated in the posterior bank of the central sulcus, corresponding to Brodmann area 3b, but it is possible that a minor contribution to the P25 component arises from a different generator located in the anterior bank of the central sulcus corresponding to the Brodmann area 4 [8, 9]. These different limitations about SSEP lead to suggest that a low amplitude threshold should not be recommended as an isolated indicator for WLST although an amplitude above the higher fixed threshold could be used to promote continued life-sustaining treatments.

Our study has several strengths. First, this is a prospective study including patients still comatose 3 days after CA, in concordance with current guidelines for neuro-prognostication and with the clinical pertinence of this evaluation. Second, SSEP recordings were assessed in a standardized way by the same technician and the interpretation was blinded to other prognostic tools and performed by the same electrophysiologist all along the study period. Moreover, measures of N20-baseline and N20–P25 amplitudes were automatized by the analysis software and were verified with a second analysis realized by the expert neurophysiologist. Third, outcomes were assessed using the best CPC scale to avoid considering patients who recovered consciousness after CA and subsequently died from non-neurological causes as poor neurological outcome (CPC 5) [18]. The CPC was assessed at 3 and 6 months permitting a long-term outcome assessment. Finally, we also evaluated the relationship between SSEP amplitudes and other demographic and prognostic markers, and we assessed the prognostic value of different combinations. Indeed, using the most specific predictors with the higher specificity and so the lowest FPR and combined predictors maximize safety of neuro-prognostication after CA [2, 28], as we highlighted in our study.

This study also has some limitations. First, this is monocentric study with a limited sample size due to the selection of patients comatose 72 h after CA. In fact, in the first 48 h after CA, many patients died because of shock, multiple organ failure or brain death, while those who recovered consciousness did not undergo neuro-prognostication. We chose this timepoint of assessment, because we believe that it corresponds to the clinical relevance of the neuro-prognostication question. Second, the timing of SSEP assessment was not strictly uniform, even if the median delay from CA was 3 days, as recommended [2]. Third, the physician in charge was not blinded to the results of N20 amplitude. Consequently, we cannot exclude that this may have affected patients’ management regarding self-fulfilling prophecy, a common bias in this setting although WLST decisions were not based on SSEP amplitudes. Fourth, we defined poor outcome as CPC 3–4–5, although some patients described as CPC 3 at 3 months could improve to a CPC 2 score at 6 months. Despite this, we collected CPC at 3 and 6 months, and we did not observed any clinical improvement over these two different times, possibly due to the limited sample size of our study. Otherwise, we only performed a visual analysis of the EEG background and nor quantitative analysis using machine learning. As visual EEG interpretation could be subjective and requires medical expertise, quantitative EEG analysis could be a promising alternative to visual analysis [29, 30]. As our study was designed to evaluate the amplitudes SSEP prognostic value and not specifically the EEG performance, we only compared SSEP prognostic performance to markers of neuro-prognostication already use in the ERC/ESICM guidelines [2]. Fifth, we cannot exclude that SSEP amplitude could vary according to the test machine parameters and recording protocols (i.e., stimulus intensity). We also used a two channel equipment (spinal response N13 being not recorded as near-field potential) and we cannot exclude that the use of this classical SSEP montage, as well as the electrodes’ location, could underestimate the real maximal amplitude on a given patient inherent to the inter individual variability of the measure. Future studies with higher electrode density systems could improve the positive predictive value for good outcome prediction. In consequence, we believe that low SSEP amplitude should not be recommended for WLST but could be used to inform families about a high probability of unfavorable neurologic outcome in case of persistent indeterminate prognostic despite use of ERC/ESICM prognostication algorithm [31]. On the other hand, amplitude above the higher threshold could be used to maintain organ support therapy due to the high probability of favorable outcome, although further large sample studies are needed to confirm these results. Finally, the association of N20 SSEP amplitudes with other prognostic markers seems to increase specificity while maintaining an acceptable sensitivity, pleading for a multimodal approach for prognostication of comatose patients after CA.

## Conclusion

In this prospective cohort of comatose patients after CA, high amplitude of cortical SSEP components, through N20-baseline and N20–P25, recorded 72 h after CA, were predictive of good outcome at 3 months, with a high specificity but a low to moderate sensitivity. Furthermore, low amplitude of N20-baseline or N20–P25 predicted poor outcome, with a higher sensitivity than the use of bilaterally absent N20. These results suggest that caution is needed regarding SSEP amplitudes in clinical routine, and that these indicators should be used in a multimodal approach for prognostication after cardiac arrest.

## Supplementary Information

**Additional file 1: ESM1**. Management protocol after CA. **ESM2**: Neurological prognostication algorithm and criteria for WLST. **ESM3**: Decision algorithm for post-resuscitation patients (Figure 1 suppl data). **ESM4**: Detailed description of the 5 categories of the Cerebral Performance Categories (CPC) Scale. **ESM5**: Scatter plot of N20-baseline and 20-P25 amplitude according to CPC at 3 and 6 months. **ESM6**: SSEP amplitudes of patients with a “AP” pattern (n=4). **ESM7**: Correlation between SSEP amplitudes and demographic characteristics or prognostics markers (Table 1 suppl data). **ESM8**: Accuracy of the different thresholds SSEP, other prognostics markers and combination for prediction of good and poor outcome (Tables 2&3 suppl data).

## Data Availability

Not applicable.

## Abbreviations

CA: Cardiac arrest
CPC: Cerebral Performance Category
EEG: Electroencephalogram
ESM: Electronic Supplementary Material
ERB: Nerve point of the neck
FPR: False Positive Rate
GCS: Glasgow Coma Scale
HIE: Hypoxic ischemic encephalopathy
ICU: Intensive-Care-Unit
IQR: Interquartile-Range
NPV: Negative Predictive Value
NSE: Neuron-specific enolase
PPV: Positive predictive value
RASS: Richmond-Agitation-Sedation-Scale
ROSC: Return of spontaneous circulation
SD: Standard Deviation
SSEP: Somatosensory evoked potentials
TTM: Targeted temperature management
WLST: Withdrawal-of-Life-Sustaining-Treatments
µV: MicroVolt
