# Supplementary material for: SSEP N20 and P25 amplitudes predict poor and good neurologic outcomes after cardiac arrest
Source: Ann Intensive Care. 2022 Mar 15;12:25. doi: 10.1186/s13613-022-00999-6 (PMC8924339; doi:10.1186/s13613-022-00999-6)
Supplement: Supplementary file 1 — Additional file 1: ESM1. Management protocol after CA. ESM2: Neurological prognostication algorithm and criteria for WLST. ESM3: Decision algorithm for post-resuscitation patients (Figure 1 suppl data). ESM4: Detailed description of the 5 categories of the Cerebral Performance Categories (CPC) Scale. ESM5: Scatter plot of N20-baseline and 20-P25 amplitude according to CPC at 3 and 6 months. ESM6: SSEP amplitudes of patients with a “AP” pattern (n=4). ESM7: Correlation between SSEP amplitudes and demographic characteristics or prognostics markers (Table 1 suppl data). ESM8: Accuracy of the different thresholds SSEP, other prognostics markers and combination for prediction of good and poor outcome (Tables 2&3 suppl data). [file 13613_2022_999_MOESM1_ESM.docx]

**SUPPLEMENTARY MATERIAL**

**SSEP N20 and P25 amplitudes predicts poor and good neurologic outcome after cardiac arrest**

Sarah Benghanem^1,2,5^, Lee S. Nguyen^3^, Martine Gavaret^2,4,7^, Julien Charpentier^1^ , Jean-Paul Mira^1,2^, Frédéric Pène^1,2,^ Angela Marchi^2,4,7^, Alain Cariou^1,2,5,6^

1. **Management protocol after CA**
2. **Neurological prognostication algorithm and criteria for WLST**
3. **Decision algorithm for post-resuscitation patients (**Figure 1 suppl data).
4. **Detailed description of the 5 categories of the Cerebral Performance Categories (CPC) Scale**
5. **Scatter plot of N20-baseline and 20-P25 amplitude according to CPC at 3 and 6 months**
6. **SSEP amplitudes of patients with a “AP” pattern (n=4)**
7. **Correlation between SSEP amplitudes and demographic characteristics or prognostics markers** (Table 1 suppl data)
8. **Accuracy of the different thresholds SSEP, other prognostics markers and combination for prediction of good and poor outcome** (Tables 2&3 suppl data).
9. **Management protocol after CA**

Immediately after OHCA and when no obviously other etiology than cardiac origin was retained, all patients had coronary angiography followed by percutaneous coronary intervention if needed. When coronary angiography was inconclusive, brain and angio-thoracic CT scan were performed to identify a reversible cause of arrest. TTM was initiated immediately at ICU admission using external cooling by forced cold air during the first 24 hours to obtain a target temperature between 32 and 34°C as recommended and subsequently rewarmed to 36°C at a rate of 0.3°C/h. Renal replacement therapy was initiated in case of severe metabolic acidosis and/or in case of life-threatening hyperkalemia, defined as a blood potassium level higher than 6 mmol.L^-1^. A mean arterial blood pressure (MAP) between 65 and 75 mmHg was targeted during the ICU stay. Post-resuscitation shock was defined as a MAP<60 mmHg or a systolic blood pressure<90 mmHg sustained for more than six hours after ROSC, despite adequate fluid loading, and requiring norepinephrine or epinephrine infusion. Clinical and non-convulsive seizures were treated with antiepileptic drugs (phenytoin, phosphenytoine, levetiracetam, valproate or phenobarbital). During the first 48 hours of ICU stay, treatments were adapted to maintain homeostasis with glucose control, normocapnia, inspired fraction of O_2_ titrated for arterial saturation of 94-98%, mean arterial pressure target to 65-70mmHg, and hemoglobin level over 7 g.dL^-1^.

Concerning sedation regimen, before 2014 we used midazolam and fentanyl with dose titrated to RASS -5 (no response to voice or physical stimulation). For midazolam the infusion starts at 4 mg.h^-1^ and the rate is increased by 1mg.h^-1^ if RASS -4 or by 2mg.h^-1^ if RASS is -3 or more after a bolus of 2 mg. Fentanyl is started at 0,7 μg.kg^-1^.h^-1^ and the titration is made with steps of 25 μg.h^-1^. When the goal is reached we systematically use vecuronium or atracurium to neuromuscular blocking according to train of four responses with 1 or 2 responses. Sedation was interrupted after rewarming and a train of four responses with 4 responses, ensuring that RASS was assessed only after clearance of NMB. In 2014, considering the high incidence of delayed awakening with midazolam-fentanyl, we have decided to change our sedation regimen towards short-acting-drugs. We used after 2014 a sedative protocol with propofol and remifentanil with dose titrated to RASS -5 (no response to voice or physical stimulation). For propofol the infusion starts at 1 mg.kg^-1^.h^-1^ and the rate is increased by 0.1 mg.kg^-1^.h^-1^ with a maximal dose of 4 mg.kg^-1^.h-^1^. Remifentanil is started at 10 μg.kg^-1^.h^-1^ and the titration is made with steps of 1 μg.kg^-1^.h^-1^ with a maximal dose of 18 μg.kg^-1^.h^-1^. When the sedation goal is reached shivering are appraised according to bedside shivering assessment scale (BSAS) every 3hours, with a goal of 0 (no shivering). If goal is not achieved a bolus of Atracurium 0.4 mg.kg^-1^ is given, after two bolus in two hours, an infusion of 0.3 mg.kg^-1^.h^-1^ is started. The titration is made with steps of 0.15 mg.kg^-1^.h^-1^. Sedation was interrupted after rewarming and a train of four responses with 4 responses, ensuring that RASS was assessed only after clearance of NMB.

1. **Neurological prognostication algorithm and criteria for WLST**

After the initial period of TTM and rewarming, neurological outcome is assessed daily in every patient by ICU physicians until death or ICU discharge. At 48 hours after discontinuation of sedation, in patients who do not awake, GCS, pupillary and corneal reflexes are assessed and an SSEP/EEG are performed. Due to inclusion period (2019-2021), we based our neuroprognostication algorithm on post resuscitation care guidelines published on 2015 (see below). When two or more of the following conditions were present: 1) bilaterally absent pupillary and corneal reflexes; 2) bilaterally absent N20 SSEP responses or 3) a refractory status epilepticus, suppression or burst suppression on EEG recording, and no clinical evidence suggested prolonged sedation, an ethic meeting between all team members (intensivists, nurses, neurologist, therapist), was hold to possibly decide WLST. Conversely, when major predictors of poor outcome were not present (i.e., patients with N20 potentials and cranial reflexes preserved, motor GCS more than 2), decisions to withhold or withdraw life-support therapies were systematically delayed in order to search for a confounding factor (sepsis, remaining sedative drug effect, inter-current disease process, other neurological disease). After this additional delay, an ethic meeting is held to incorporate all prognostic variables in the decision. This decision could be either to withhold or withdraw life-support therapies.

WLST was always decided after a collegial decision. All deaths associated with end-of-life decisions occurred during the ICU stay.

**CA with coma**

**Discontinuation of sedation 48h**

**TTM**

**Neurologic examination :**

**- Comatose patients with GCS motor < 3 and a RASS ≤-4**

**2 criteria among:**

**- Bilaterally absent of pupillary and corneal reflex**

**- Bilaterally absent N20 SSEP waves**

**- EEG: Refractory status epilepticus, Suppression or burst suppression**

**Withdrawal of life-sustaining treatments**

**Neurologic assesment**

**Continue ICU treatment**

**Reevaluate daily**

**No**

**Yes**

**No**

**Yes**

**Ethic conference**

**Figure 1 suppl data.** Decision algorithm for post-resuscitation patients Adapted from Nolan et al, Resuscitation and ICM 2015[14]

1. **Detailed description of the 5 categories of the Cerebral Performance Categories Scale (CPC)** *(Edgren E, Hedstrand U, Kelsey S, Sutton-Tyrrell K, Safar P (1994) Assessment of neurological prognosis in comatose survivors of cardiac arrest. BRCT I Study Group. Lancet 343:1055-1059.)*[17]**:**

CPC 1: Good cerebral performance (normal life: Conscious, alert, able to work and lead a normal life; may have minor psychological or neurologic deficits (mild dysphasia, non-incapacitating hemiparesis, or minor cranial nerve abnormalities).

CPC 2: Moderate cerebral disability (disabled but independent): Conscious, sufficient cerebral function for part-time work in sheltered environment or independent activities of daily life (dress, travel by public transportation, food preparation); may have hemiplegia, seizures, ataxia, dysarthria, dysphasia, or permanent memory or mental changes.

CPC 3: Severe cerebral disability (conscious but disabled and dependent): Conscious, dependent on others for daily support (in an institution or at home with exceptional family effort); has at least limited cognition. This category includes a wide range of cerebral abnormalities, from patients who are ambulatory but have severe memory disturbances or dementia precluding independent existence to those who are paralyzed and can communicate only with their eyes, as in the locked-in syndrome.

CPC 4: Coma/vegetative state (unconscious): Unconscious, unaware of surroundings, no cognition. No verbal or psychological interaction with environment.

CPC 5: death (certified brain dead or dead by traditional criteria).

1. **Scatter plot of N20-baseline and N20-P25 amplitude according to CPC at 3 and 6 months**


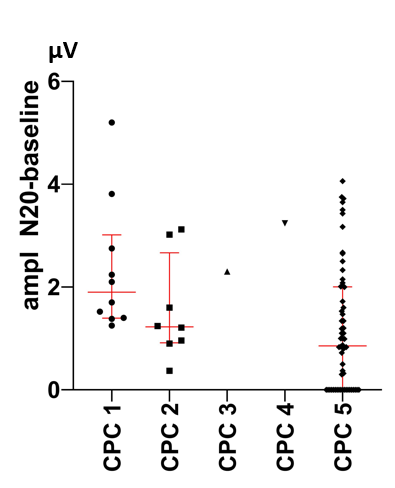


**a. Scatter plot of N20-baseline amplitudes according to CPC at 3 months**


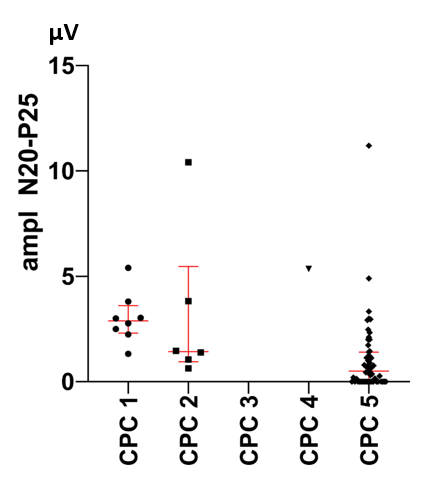


**b. Scatter plot of N20-P25 amplitudes according to CPC at 3 months. Missing data n=3.** Amplitudes of somatosensory evoked potentials (SSEPs) largely overlap for the 5 outcome groups.


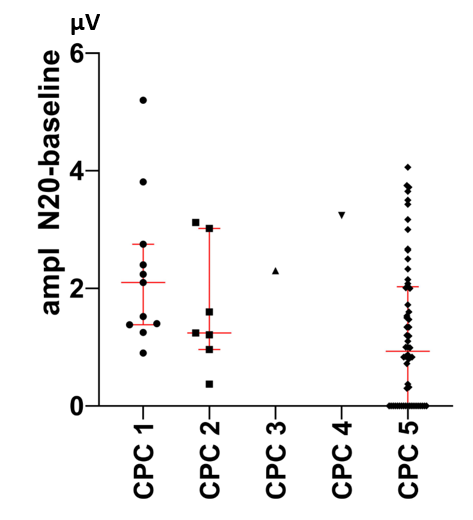


**c.** Scatter plot of N20-baseline amplitudes **according to CPC at 6 months**


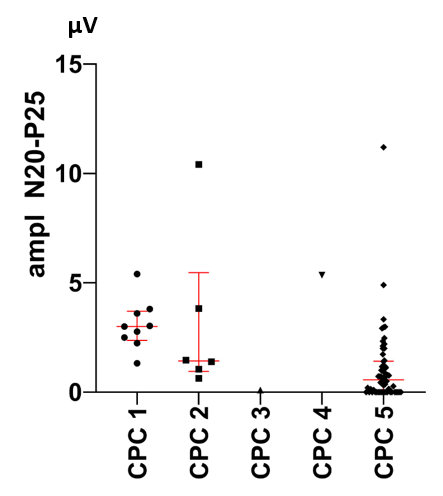


**d.** Scatter plot of N20-P25 amplitudes **according to CPC at 6 months. Missing data n=3.** Amplitudes of somatosensory evoked potentials (SSEPs) largely overlap for the 5 outcome groups.

1. **SSEP amplitudes of patients with a “AP” pattern (n=4)**

- **N20-baseline =1.34 µV**
- **N20-P25= 0.72 µV**

1. **Correlation between SSEP amplitudes and demographic characteristics or prognostics markers.**

|  | | | **N20-baseline amplitudes**  Correlation coefficient  r | **N20-P25 amplitudes**  Correlation coefficient  r |
| --- | --- | --- | --- | --- |
|  |  | **Low_flow** | -0.12 | **-0,26*** |
|  |  | **NSE Day 2** | **-0.27^*^** | **-0,47**** |
|  |  | **NSE Day 3** | **-0.37^**^** | **-0,54**** |
|  |  | **Benign EEG** | 0.38******* | **0.36****** |
|  |  | **No status myoclonus** | 0.17 | 0.05 |

***p=0.05 **p<0.01** ***p=0.06 ******p=0.02**

**Table 1 suppl data.** Correlation between SSEP amplitudes and demographic characteristics or prognostics markers.

**7.** **Accuracy of the different thresholds SSEP, other prognostics markers and combination for prediction of good and poor outcome**

|  | **Se(%)** | **Sp(%)** | **FPR(%)** |
| --- | --- | --- | --- |
| **N20-baseline > 4,6µV** | **5(3-7)** | **100(100-100)** | **0(0-0)** |
| **N20- baseline >2.7µV + benign EEG** | **22.2(18-27)** | **97(95-99)** | **3(1-5)** |
| **N20- baseline >2.7µV + NSE< 60ng/ml** | **16.7(13-21)** | **95.3(93-98)** | **4.7(2-7)** |
| **N20- baseline >2.7µV + no status myoclonus** | **22.2(18-27)** | **93.8(91-96)** | **6.3(4-9)** |

**Table 2 suppl data.** Accuracy of SSEP amplitudes, EEG patterns, no myoclonus, NSE peak at day 3 <60 µg/ml, and combination for prediction of good outcome

|  | **Se(%)** | **Sp(%)** | **FPR(%)** |
| --- | --- | --- | --- |
| **N20-baseline< 0,88µV + status myoclonus** | **27(22-31)** | **100(100-100)** | **0(0-0)** |
| **N20-P25< 1µV + status myoclonus** | **33(28-38)** | **100(100-100)** | **0(0-0)** |
| **N20-baseline< 0,88µV + malignant EEG** | **22(17-26)** | **100(100-100)** | **0(0-0)** |
| **N20-P25< 1µV + malignant EEG** | **30(25-35)** | **100(100-100)** | **0(0-0)** |

**Table 3 suppl data**. Accuracy of the SSEP and EEG patterns, status myoclonus, NSE>60 µg/ml and combination for prediction of poor outcome
